# Rapid Cerebral Metabolic Shift during Neonatal Sepsis Is Attenuated by Enteral Colostrum Supplementation in Preterm Pigs

**DOI:** 10.3390/metabo9010013

**Published:** 2019-01-11

**Authors:** Masoumeh Alinaghi, Ping-Ping Jiang, Anders Brunse, Per Torp Sangild, Hanne Christine Bertram

**Affiliations:** 1 Department of Food Science, Aarhus University, Kirstinebjergvej 10, 5792 Aarslev, Denmark; masoumeh.alinaghi@food.au.dk; 2 Comparative Pediatrics and Nutrition, Department of Veterinary and Animal Sciences, Faculty of Health and Medical Sciences, University of Copenhagen, 1870 Frederiksberg, Denmark; pipi@sund.ku.dk (P.-P.J.); anderss@sund.ku.dk (A.B.); pts@sund.ku.dk (P.T.S.); 3 School of Public Health, Sun Yat-sen University, 510220 Guangzhou, China

**Keywords:** NMR metabolomics, neonatal sepsis, preterm infants, bloodstream infection, cerebrospinal fluid, *Staphylococcus epidermidis*, brain metabolites, cerebral metabolism, enteral feeding, bioactive dairy components

## Abstract

Sepsis, the clinical manifestation of serious infection, may disturb normal brain development, especially in preterm infants with an immature brain. We hypothesized that neonatal sepsis induces systemic metabolic alterations that rapidly affect metabolic signatures in immature brain and cerebrospinal fluid (CSF). Cesarean-delivered preterm pigs systemically received 10^9^ CFU/kg *Staphylococcus epidermidis* (SE) and were provided total parenteral nutrition (*n* = 9) or enteral supplementation with bovine colostrum (*n* = 10) and compared with uninfected pigs receiving parenteral nutrition (*n* = 7). Plasma, CSF, and brain tissue samples were collected after 24 h and analyzed by ^1^H NMR-based metabolomics. Both plasma and CSF metabolomes revealed SE-induced changes in metabolite levels that reflected a modified energy metabolism. Hence, increased plasma lactate, alanine, and succinate levels, as well as CSF lactate levels, were observed during SE infection (all *p* < 0.05, ANOVA analysis). *Myo*-inositol, a glucose derivative known for beneficial effects on lung maturation in preterm infants, was also increased in plasma and CSF following SE infection. Enteral colostrum supplementation attenuated the lactate accumulation in blood and CSF. Bloodstream infection in preterm newborns was found to induce a rapid metabolic shift in both plasma and CSF, which was modulated by colostrum feeding.

## 1. Introduction

Sepsis, the clinical manifestation of an uncontrolled bloodstream infection (BSI), is a frequent cause of morbidity and mortality in newborns, especially in preterm infants with very low birth weight [1]. Coagulase-negative staphylococci are among the most frequent pathogens in neonatal sepsis, particularly *Staphylococcus epidermidis* (SE), which is often acquired from the hospital environment through invasive medical devices [2]. Preterm infants born at a lower gestational age are most susceptible to hospital-acquired infections and life-threatening sepsis due to their immature innate immune system and limited maternal passive immunity [3].

In recent decades, the incidence of neonatal sepsis related to hospital-acquired infections has increased together with improved survival of the majority of preterm infants due to advances in medical care [1,4,5]. Nevertheless, neonatal sepsis increases the risk of brain injury and neurodevelopmental impairment [6,7]. An elevated level of proinflammatory cytokines, as part of the infection-related inflammatory response, is neurotoxic and increases the permeability of the blood–brain barrier and may cause neurodevelopmental impairments [8,9]. Thus, exposure to neonatal systemic infection leads to central nervous system (CNS) inflammation, potentially causing brain injury with long-lasting consequences for neurological and mental health [6,10]. It remains unclear if the infection-induced systemic inflammatory reactions are also associated with rapid metabolic perturbations in the cerebrospinal fluid (CSF) and the immature brain. It has been shown that endogenous metabolites, such as succinate and itaconate, may play separate roles to regulate inflammatory responses [11,12]. In addition, increases in the intracellular glycerophosphoinositol level of macrophages can be observed in lipopolysaccharide-induced inflammation, probably reflecting a modulatory effect on inflammatory responses [13]. Systemic metabolic adaptations during neonatal sepsis have been demonstrated [14] but the effect on cerebral energy metabolism is less known, though it may be of importance.

Diminishing BSI and systemic inflammation may reduce the risk of brain injury and improve long-term outcomes in preterm infants [8,9]. Human milk feeding decreases the risk of sepsis in preterm infants [15]. Colostrum, the first milk of mammals, contains a multitude of anti-inflammatory and anti-microbial proteins and peptides, known to protect the immature intestine of preterm infants from opportunistic enteric bacteria and the development of necrotizing enterocolitis (NEC) [16,17,18,19,20,21]. Whether colostrum feeding can also ameliorate systemic inflammation and protect against BSI and the associated metabolic effects in preterm infants remains to be clarified. Using a clinically relevant BSI model in preterm pigs, we previously showed that SE inoculation caused a severe inflammatory response in CSF and plasma and that enteral colostrum supplementation reduced sepsis incidence and brain inflammation [16]. Building further on these initial findings, we hypothesized that SE infection induces metabolic perturbations in the immature brain and CSF, which may relate to perturbations in blood plasma. Thus, we investigated if neonatal BSI changes the plasma, CSF, and brain tissue metabolomes as part of the inflammatory response and if early colostrum feeding modulates the metabolome. Metabolomics is a relevant tool to describe the metabolic perturbations and adaptations in body fluids which accompany infection and inflammation [22,23,24]. Thus, ^1^H-NMR and high-resolution magic-angle spinning (HR-MAS) NMR spectroscopic analyses were adopted to profile the metabolomes of CSF, plasma, and brain.

## 2. Materials and Methods

### 2.1. Animal Experimental Procedures

Twenty-six pigs from two sows (Danish landrace × Large White × Duroc) were delivered by cesarean section at 90% gestation. Shortly after birth, animals were transferred to individual preheated (37 °C) incubators with oxygen supply (1–2 L/min) and fitted with umbilical arterial catheters and orogastric feeding tubes. The pigs were stratified by sex and birth weight and randomly allocated to one of three groups: (i) a control group receiving intra-arterial saline and 6 mL/kg/h total parenteral nutrition, TPN (CON + TPN, *n* = 7), (ii) an infected group receiving SE inoculation (1.0 × 10^9^ CFU/kg body weight) and 6 mL/kg/h TPN (SE + TPN, *n* = 9), or (iii) a colostrum supplementation group receiving the same dose of SE and 3 mL/kg/h supplementary parenteral nutrition + 10 mL/kg/3 h bovine colostrum (SE + COL, *n* = 10). The bovine colostrum (Biofiber-Damino, Gesten, Denmark) was produced from the first and second milkings within 24 h after the parturition of Danish Holstein dairy cattle, Holder-pasteurized and spray-dried before use. Animals were administered SE or saline by a 3-min continuous intra-arterial infusion within 4 h after birth. Detailed descriptions of nutritional compositions of colostrum and parenteral formulation and preparation of the SE inoculate are available elsewhere [25,26]. Twenty-four hours after SE administration, a mixed blood sample was collected by intracardial puncture to obtain plasma, and piglets were subsequently euthanized with a lethal intracardial injection of barbiturate. CSF was collected by suboccipital puncture and the brains were gently collected. Six brain structures of interest (cerebellum, periventricular white matter (PVMW), striatum, hypothalamus, prefrontal cortex, and midbrain) were dissected and stored at −80 °C until analysis. In order to investigate the correlation between the acquired metabolomics data and neuroinflammation, we re-analyzed previously published cerebral immune cell staining and hippocampus gene expression data obtained from the same animals as the present metabolomics study. A detailed description of the methodologies as well as the 24-h survival rate can be found elsewhere [25]. The animal procedures were all approved by the Danish National Committee on Animal Experimentation (2014-15-0201-00418).

### 2.2. NMR Sample Preparation

#### 2.2.1. Liquid ^1^H NMR Spectroscopy

Thawed plasma samples were filtered using Amicon Ultra 0.5-mL 3 kDa (Millipore, Billerica, MA, USA) and spin filtering at 10,000 × *g* at 4 °C for 3 h to remove the lipid and proteins. No filtration was performed on the CSF samples. A mixture of 100 µL CSF sample, 490 µL D_2_O, and 10 µL D_2_O containing 0.05% sodium trimethylsilyl-[2,2,3,3-^2^H_4_]-1-propionate (TSP; Sigma-Aldrich, St. Louis, MO, USA) as an internal chemical shift reference was transferred to a 5-mm NMR tube (Wilmad, Vineland, NJ, USA). For plasma samples, a mixture of 400 µL filtered samples, 100 µL phosphate buffer in D_2_O [27] (pH = 7.4), and 100 µL D_2_O containing 0.05% TSP were added into an NMR sample tube.

#### 2.2.2. HR-MAS ^1^H NMR Spectroscopy

Frozen brain tissues were cut on a dry-ice bench to fit into 30-µL HR-MAS rotor inserts. D_2_O containing 0.05% TSP was added into the inserts to provide a field-frequency lock for the NMR spectrometer. The inserts were placed in 4-mm zirconium rotors for NMR analysis.

### 2.3. NMR Data Acquisition and Preprocessing

NMR spectra were recorded on a Bruker Avance III 600 MHz NMR spectrometer (Bruker BioSpin Gmbh, Rheinstettten, Germany) equipped with a 5-mm ^1^H TXI probe for fluid samples and a 5-mm HR-MAS probe for intact tissues operating at a ^1^H frequency of 600.13 MHz. A target temperature of 298 K (CSF and plasma) and a relaxation delay of 5 s were applied. A total of 256 and 128 free induction decays (FIDs) were acquired for the CSF and plasma samples, respectively. The acquisition parameters included 32,768 data points, a spectral width of 7288 Hz, and an acquisition time of 2.25 s. One-dimensional nuclear Overhauser effect spectroscopy (1D-NOESY) experiment with a single 90° pulse sequence and pre-saturation of the water resonance was conducted. 

HR-MAS ^1^H NMR spectroscopy was carried out at 281 K with a spinning speed of 5000 Hz. 1D-NOESY experiment with pre-saturation was performed using a recycling delay of 3 s with an acquisition time of 2.25 s. A spectral width of 7288 Hz was employed. In addition, the Carr–Purcell–Meiboom–Gill (CPMG) pulse sequence with a T_2_ filter of 400 ms was applied. The acquisition parameters included 128 scans, 32K data points, and a spectral width of 10,417 Hz using a recycling delay of 3 s with an acquisition time of 1.57 s.

The spectra were processed with zero-filling prior to Fourier transformation. All spectra were referenced to the TSP signal at 0.0 ppm. An experimental window function with a line-broadening factor of 0.3 Hz was applied to all FIDs before Fourier transformation. The resulting spectra were manually phase- and baseline-corrected by polynomial using the Topspin^TM^ 3.0 software (Bruker BioSpin, Gmbh, Rheinstetten, Germany). The NMR resonances were assigned based on two-dimensional (2D) NMR spectroscopy, the Human Metabolome Database [28], existing literature [29,30,31,32,33], and Chenomx NMR Suite 7.7 (Chenomx Inc, Edmonton, AB, Canada).

### 2.4. Data Analysis

Matlab (version R2016b, MathWorks Inc., MA, United States) was used for data analysis and visualization. Using the interval-correlation-shifting algorithm (icoshift) [34], the NMR spectra were aligned by local shifting of a total number of 50 intervals and customized intervals for CSF, plasma (with five missing samples from the total of 26 piglets, CON + TPN (*n* = 7), SE + TPN (*n* = 5), and SE + COL (*n* = 9)), and brain tissue data (with four missing samples from the total of 26 piglets, CON + TPN (*n* = 7), SE + TPN (*n* = 5), and SE + COL (*n* = 10)). Data were normalized to the intensity of the TSP signal, and the brain data were further normalized to the total sum area under the peak. The spectral region containing the residual water resonance (4.7–5.00 ppm) was excluded. Moreover, unassigned peaks at 0.72 (t), 0.83 (t), and 0.95 (d) ppm were observed in the ^1^H NMR spectra of the CSF samples. Inspection of the 2D ^1^H-^13^C spectra showed that the ^1^H resonances correlate to ^13^C resonances at 11.83, 16.12, and 16.44 ppm, but assignment was not possible using available databases. These unassigned spectral regions and regions containing signals from ethanol with resonances at 1.20 (t) and 3.60 (q) ppm and propylene glycol at 1.10 (d), 3.40 (dd), 3.50 (dd), and 3.90 (m) ppm were excluded from the NMR spectra of the CSF samples prior to further analyses. High concentrations of ethanol can be the result of contamination during sampling [35]. In HR-MAS brain data, only the aliphatic part (1.3–4.5 ppm), excluding the ethanol and propylene glycol resonances, was used for further analysis, as the aromatic part of the spectra contained few signals and therefore mostly contributed to noise. CSF, plasma, and brain tissue NMR spectra were subdivided into 0.01 ppm bins, reducing the spectra into 908, 953, and 322 separate variables, respectively. Moreover, unrelated variation associated with a litter effect was removed by using the unbalanced ANOVA-simultaneous component analysis (ASCA) model [36]. The data were mean-centered and pareto-scaled prior to multivariate analysis. Principal component analysis (PCA) was performed to identify variations in metabolite profiles among the samples. To determine whether the clinical data correlated with the metabolic profiles, partial least squares (PLS) regressions were performed. The quality of the model was evaluated by the goodness-of-fit parameter R^2^ and the predictive ability parameter Q^2^ using the leave-one-out validation. 

In addition, quantitative analysis of the ^1^H NMR spectra was performed by the integration of peak areas using Topspin^TM^ 3.0 software (Bruker BioSpin, Gmbh, Rheinstetten, Germany). For blood and CSF, the identified metabolites were quantified based on the known internal TSP concentration. The identified metabolites of brain were normalized to the total sum area under the peak prior to analysis. A two-way ANOVA for an unbalanced design with the fixed effect of treatment, litter, and treatment × litter was used to calculate the significance level of differences in metabolite concentrations using the statistics and machine learning toolbox in Matlab. A Tukey–Kramer test with a significance level of 0.05 was performed for all pairwise comparisons. Moreover, correlations between quantified plasma metabolites and gene expression data were calculated and visualized by heat map plots. For visualization, Pearson’s correlation coefficients and *p*-values for correlation between variables were evaluated. Hierarchical cluster analysis (HCA) was initially applied to re-order the metabolites and genes in the heat map according to similarities between the variables.

## 3. Results

**Figure 1:**
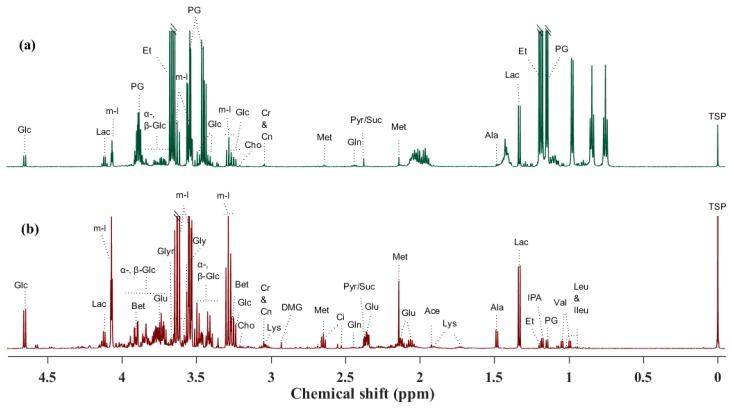
^1^H NMR spectra (obtained at 600 MHz) of a control sample and the identified metabolites: (**a**) cerebrospinal fluid (CSF) and (**b**) plasma. Abbreviations: Ace: acetate; Ala: alanine; Bet: betaine; Cho: choline; Ci: citrate; Cn: creatinine; Cr: creatine; DMG: N,N-dimethylglycine; Et: ethanol; α-, β-Glc: α-, β-glucose; Glu: glutamate; Gln: glutamine; Glyr: glycerol; Gly: glycine; Ileu: isoleucine; IPA: isopropanol; Lac: lactate; Leu: leucine; Lys: lysine; Met: methionine; m-I: *myo*-inositol; PG: propylene glycol; Pyr: pyruvate; Suc: succinate; Val: valine.

**Figure 2:**
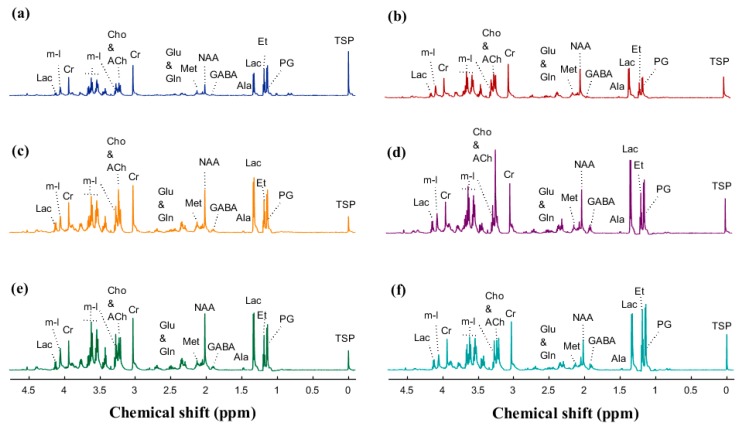
High-resolution magic-angle spinning (HR-MAS) ^1^H NMR spectra (obtained at 600 MHz) of control brain samples and the identified metabolites: (**a**) cerebellum, (**b**) periventricular white matter, (**c**) striatum, (**d**) hypothalamus, (**e**) prefrontal cortex, and (**f**) midbrain. Abbreviations: Ala: alanine; ACh: acetylcholine; GABA: γ-aminobutyric acid; Cho: choline; Cn: creatinine; Cr: creatine; Et: ethanol; Glu: glutamate; Gln: glutamine; Lac: lactate; Met: methionine; m-I: *myo*-inositol; NAA: N-acetylaspartate; PG: propylene glycol.

The baseline characteristics birth weight (935, 1030, and 944 g) and male gender frequency (57, 44, and 40%) were not significantly different between CON + TPN, SE + TPN, and SE + COL groups by one-way ANOVA and Fisher–Freeman–Halton exact test, respectively. CSF, blood plasma, and six brain areas of interest from preterm newborn pigs were analyzed by ^1^H NMR and HR-MAS NMR spectroscopy, and a total of 31, 36, and 26 metabolites were assigned in CSF, plasma, and brain tissues, respectively (Figure 1 and Figure 2). 

**Figure 3:**
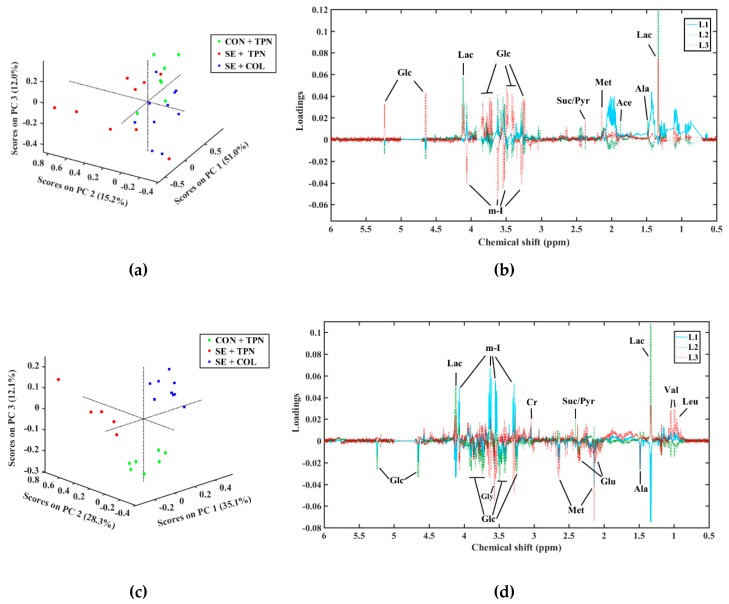
Principal component analysis (PCA) score and loading plots of the NMR data of (**a**,**b**) CSF and (**c**,**d**) plasma samples. CON + TPN (green diamond) represents the control group, SE + TPN (red circles) represents the infected group by *Staphylococcus epidermidis* (SE), and SE + COL (blue squares) represents the colostrum (COL) supplementation group. In the loading plots: Ace: acetate; Ala: alanine; Cr: creatine; Glc: glucose; Glu: glutamate; Gly: glycine; Lac: lactate; Leu: leucine; Met: methionine; m-I: *myo*-Inositol; Pyr: pyruvate; Suc: succinate; Val: valine. L1, L2, and L3 are the first, second, and third loadings, respectively.

A PCA score plot of the CSF metabolome revealed a tendency for separation of the SE + TPN group from the other two groups along the second principal component (PC2), while the SE + COL and control samples were grouped along PC3 (Figure 3a,b). Elucidation of the loading plot showed that separation of the SE + TPN group could be ascribed mainly to an increase in lactate, alanine, and *myo*-inositol levels, and a decrease in the glucose level in the SE + TPN group. 

A two-way ANOVA was calculated for metabolites contributing to the grouping in the PCA score plots, which revealed significant differences (*p* < 0.05) among treatment groups for lactate levels with a higher concentration in the SE + TPN group and a reduced level after COLsupplementation (Table 1). The litter and treatment × litter *p*-values can also be found in Table S1 (Supplementary Materials). 

**Figure 4:**
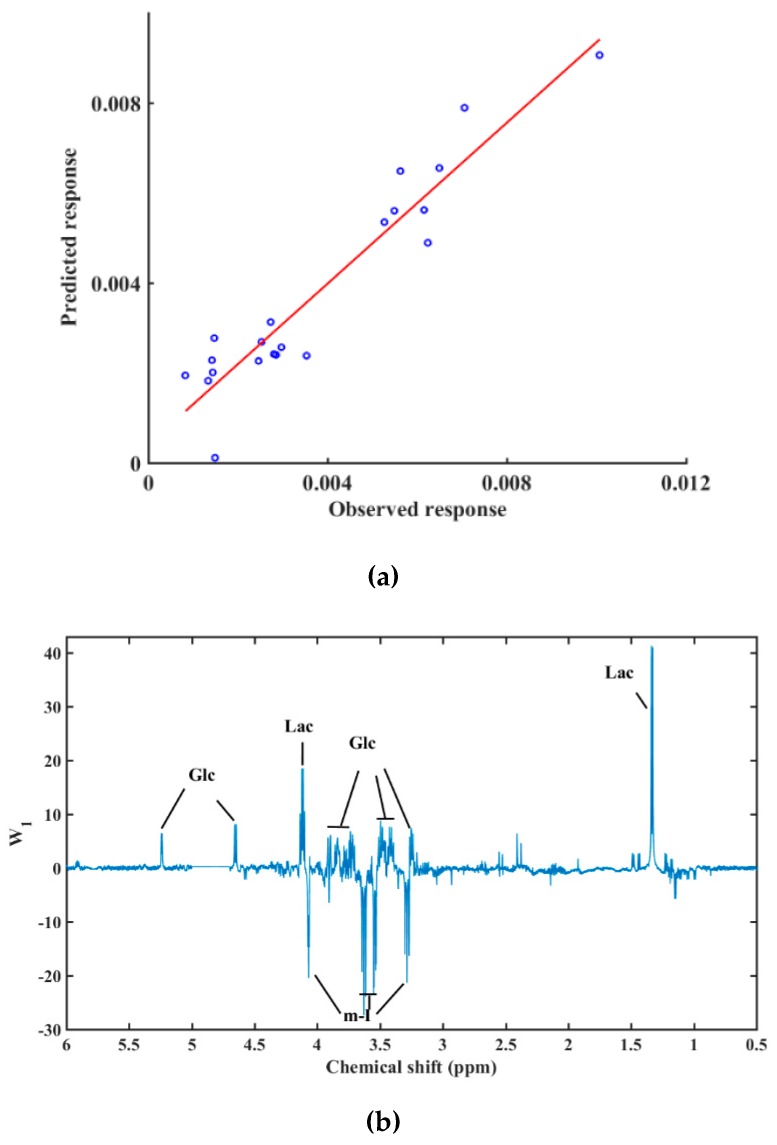
Results from the partial least squares (PLS) regression model between the plasma NMR spectra (X-variables) and cerebral microglia density (y-variable) obtained by three components, validated by the leave-one-out method (R^2^ = 0.89, Q^2^ = 0.55, and root mean square prediction error (RMSPE) = 1.6 × 10^−3^). (**a**) Predicted versus measured cerebral microglia density, (**b**) PLS weights revealing NMR variables that are important predictors. Abbreviations: Glc: glucose; Lac: lactate; m-I: *myo*-Inositol.

Results from the plasma metabolome showed the same trend as metabolite perturbations identified in CSF, and the PCA score plot revealed a strong separation between treatment groups (Figure 3c,d). The SE + TPN group had higher levels of plasma lactate, alanine, and succinate/pyruvate (all *p* < 0.05, Figure 3d and Table 1) compared with CON + TPN and SE + COL. Furthermore, PCA loadings revealed that resonances from glucose and *myo*-inositol exhibited decreased and increased intensities in SE + TPN piglets, respectively, and contributed to the group separation, although not reaching a statistically significant difference between groups in the ANOVA analysis, possibly due to the small samples size and the small nature of the differences (Table 1). In addition, the SE + COL group was separated from the CON + TPN group along PC3 with lower levels of plasma methionine and higher levels of leucine and valine in SE + COL (all *p* < 0.05, Figure 3b and Table 1), possibly due to an absorption and metabolism of colostrum proteins. A PLS model with three components (R^2^ = 0.89, Q^2^ = 0.55, and root mean square prediction error (RMSPE) = 1.6 × 10^−3^) indicated that the NMR spectra of plasma had a good fit and prediction ability for the cerebral microglia density (Figure 4a). From the PLS weights, it could be observed that lactate and *myo*-inositol were the most important variables to build a quantitative relationship between the NMR data and cerebral microglia density (Figure 4b). 

**Figure 5:**
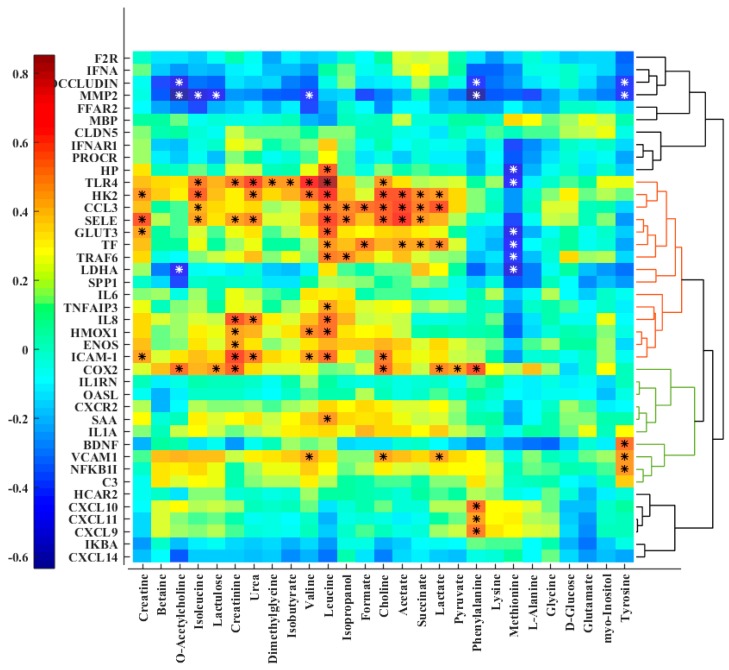
Heat map of correlation analysis between the quantified plasma metabolites and hippocampal gene expression data. The genes were clustered and re-ordered based on the correlation in their expression profiles by hierarchical cluster analysis (HCA). Stars indicate significant correlations (*p* < 0.05).

Correlation analysis between plasma metabolites and hippocampus gene expression data revealed a positive correlation of expression levels of some genes (mostly clustered together and presented as colored HCA on the right-hand side of the heat map), while genes such as *HK2*, *TLR4*, *CCL3*, *SELE*, and *TF* as well as *COX-2* (also known as *PTGS2*), *ICAM1* and *VCAM1* were significantly correlated with lactate, succinate, and choline (Figure 5). These genes were upregulated in the sepsis condition, while the expression levels of *COX-2* and *SELE* were reduced after colostrum supplementation [25]. 

For metabolomes of various brain parts, a clear separation of the treatment groups could not be observed in the PCA score plots (data not shown). However, among the different brain parts, PVMW showed a tendency toward the clustering of treatment groups and choline-containing metabolites contributed mostly to the variation in the data (Figure S1 in Supplementary Materials). Two-way ANOVA of the metabolites responsible for the variation in the data revealed that the intensity of choline was different (*p* = 0.06) among treatment groups with a lower level in the SE + TPN group and an increased level after COL supplementation (Table 1). On the other hand, the opposite trend could be observed in the plasma metabolome where choline had a significantly higher concentration in the SE + TPN samples (*p* < 0.05).

## 4. Discussion

In the present study, we examined the perturbations in systemic and cerebral metabolism and the impact of a bioactive milk diet, bovine colostrum, during BSI in a preterm pig model. After 24 h of SE administration, NMR-based metabolomics revealed significant changes in the plasma level of key intermediates involved in energy metabolism, such as lactate, alanine, and succinate as well as specific amino acids. These results are in agreement with other studies showing that sepsis causes impaired cerebral energy metabolism and disturbances in the tricarboxylic acid (TCA) cycle [37,38]. Importantly, this metabolic alteration was mirrored in the CSF in this early phase of infection, and lactate, which is one of the most important biomarkers for impaired energy metabolism, increased significantly in infected animals and was modulated by early feeding with bovine colostrum.

**Figure 6:**
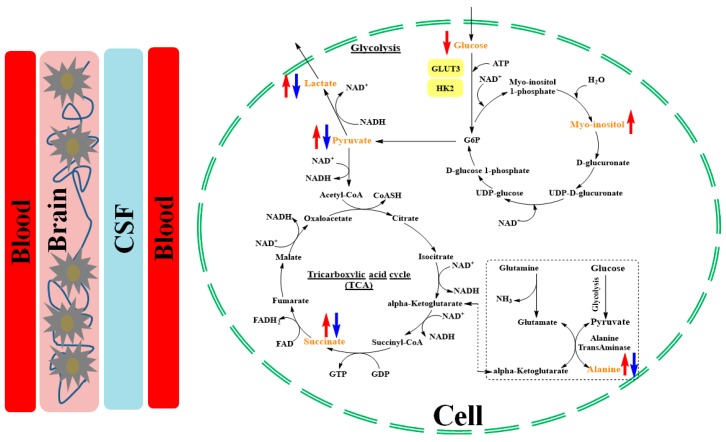
Schematic representation of altered metabolites during systemic inflammatory response under BSI condition. Blood delivers the glucose to tissues, where it reaches cells through diffusion and by glucose transporters. Glucose is converted to G6P and then pyruvate through glycolysis, leading to lactate accumulation as well as an elevated tricarboxylic acid cycle. The altered metabolites are shown in yellow. Red and blue arrows represent SE + TPN and SE + COL, respectively, and denote higher and lower levels of the respective metabolites compared to controls.

Alterations in lactate levels revealed effects on energy metabolism associated with an enhanced glycolysis during BSI. This enhanced glycolysis appears to be linked with an upregulation of hypoxia-regulated genes in the hippocampus, when aerobic energy metabolism is inhibited by decreased oxygen availability and a high demand of energy during infection [39]. The decreased glucose level during BSI indicates either an increased glucose consumption by circulating bacteria or an increased cellular uptake and conversion to glucose-6-phosphate (G6P) [39]. Due to limited oxygen availability, the electron transport chain stalls, electron acceptors, cannot be regenerated, causing succinate levels to increase. To increase the production of adenosine triphosphate, the main source of cellular energy, glycolysis is enhanced, leading to increased levels of pyruvate, which is converted to either lactate to regenerate glycolytic electron acceptors or to alanine, a gluconeogenic substrate. An overview of the altered metabolic pathways is illustrated in Figure 6. 

A significantly increased level of plasma choline was observed in infected piglets, which was attenuated after colostrum supplementation. On the other hand, infected piglets exhibited a lower level of choline in the brain. An increase in the plasma level of choline, which is a metabolite that is fundamental for the integrity of cellular membranes, lipid metabolism, and the development of the brain, can possibly be associated with the severity of the cerebral damage. Such increases in the plasma of animals with sepsis [40] as well as in the umbilical cord serum of neonatal asphyxia [41] have been reported and explained by an enhanced lipolysis and dysfunctional process of lipid metabolism [42]. On the contrary, a decreased level of choline species has been reported in the plasma of sepsis patients when compared with healthy controls [43]. However, the metabolite responses related to an enhanced energy metabolism might be different from other age groups as the studied group was encountering infection for the first time and lacked proper immune responses [44].

Besides the metabolic perturbations, hippocampus gene expression has been proposed as informative for an inflammatory condition [45]. Several studies have shown the gene expression plays an important role in regulating metabolism in response to perturbations [46,47]. A cell can be adapted to the changing environment by alteration in the expression of genes. Investigating the association between the gene expression and metabolite shifts is particularly relevant as the metabolome can reflect responses to environmental perturbation and the metabolic phenotype [46]. Linking the set of the expressed genes to metabolism, the plasma metabolome was used for a correlation analysis, since plasma can act as an internal indicator of the changes while covering a higher number of metabolites with better representation of the BSI condition as compared to other biofluids. Investigating the network of metabolite and gene interactions illustrated expression changes related to the metabolite pathway perturbations. Even though some correlations did not reach the significance level (*p* < 0.05), the establishment of a correlation pattern suggested that a proportion of the metabolite adaptation is transcriptionally regulated. A change in cerebral energy metabolism during BSI coincided with an upregulation of the glucose transporter 3 (*GLUT3*) and the rate-limiting glycolytic enzyme hexokinase 2 (*HK2*), which phosphorylates glucose to produce G6P. *TLR4* activation, which is responsible for innate immune signaling and pro-inflammatory responses, shifts the energy metabolism of circulating immune cells from mitochondrial oxidative phosphorylation to glycolysis [48,49]. On the other hand, it has been reported that an increase in succinate activates the hypoxia inducible factor-1a and consequently promotes the inflammatory gene expression [50]. 

Although a few studies have examined the effect of bovine colostrum on cytokine production [51,52], investigations on the specific metabolic impact of colostrum intervention in preterm infants are lacking. Anti-inflammatory and anti-microbial proteins and peptides in colostrum might play an important role in preventing BSI, by mechanisms such as limiting bacterial overgrowth and translocation in the gut [21]. Bovine lactoferrin intervention indicated the possibility of the prevention of the late-onset sepsisin preterm infants [53]. However, it has been reported that casein is less digestible than whey protein due to its easy coagulation in the stomach [54]. Notably, the present study revealed that colostrum supplementation attenuated the BSI-induced increases in lactate, alanine, and succinate levels in plasma with almost similar trends in CSF. This is in line with published data from the same animal experiment, showing reduced blood oxygen partial pressure and saturation during SE infection, which were prevented by colostrum supplementation [25]. Moreover, previously documented improvements in blood and CSF bacterial clearance after colostrum supplementation suggest that changes to immune activation and function may underlie the metabolic changes seen here [25]. 

In addition to the effects on metabolic intermediates involved in energy metabolism, the present study also identified significant effects of colostrum supplementation on plasma levels of methionine, valine, and leucine as well as the level of methionine in CSF. These effects may reflect direct differences in amino acid quantity and absorption between bovine colostrum and the parenteral formulation, and it remains unknown if changes in the levels of these amino acids exert a biological effect.

Effects of BSI on *myo*-inositol metabolism were also identified. *Myo*-inositol is synthesized from glucose via G6P (the first product of glycolysis) [55] (Figure 6). The concentration of *myo*-inositol increases with glial proliferation or the increase of cell size, both of which occur during inflammation [56]. Of note, we previously showed that SE infection increases both cerebral microglia/macrophage numbers and cell size [25]. In this study, a correlation between the plasma metabolome, especially lactate and *myo*-inositol, and cerebral microglia density was observed by a PLS regression model (Figure 4). Free *myo*-inositol is present in all human tissues, but its concentration is highest in newborns, suggesting an important role in the neonatal period [57], wherein serum levels have also been found to be linked with birth weight [58]. Indeed, *myo*-inositol has beneficial effects on pulmonary function in preterm infants with respiratory distress [59]. We speculate that *myo*-inositol may act to improve pulmonary function during sepsis. 

The present study used a preterm piglet model, which has formerly been shown to be a clinically relevant BSI model [16]. This piglet model has a fundamental advantage as it enables multiple samplings from different organs and tissues that would not be available in a human setup. However, the model also has its limitations. Our study revealed a considerable variation in the metabolome of the piglets even within the same treatment groups. This large inter-individual variation reflects differences in the metabolic phenotype among the piglets as well as variations in the response to the applied *S. epidermis* infection. The presence of this variation influences the ability to identify and establish generic metabolic markers. Further studies targeted at identifying the phenotypic characteristics of the piglet model are warranted.

## 5. Conclusions

Applying metabolomics analyses on a piglet model of BSI revealed associations between infection and metabolic changes related to the glycolysis and tricarboxylic acid cycle. In addition, the plasma and CSF level of *myo*-inositol was affected by BSI, which may have an impact on the lung maturation of preterm infants and pulmonary function during sepsis. Finally, the study revealed that hypoxia-related changes in systemic and cerebral energy metabolism were attenuated by oral COL supplementation, suggesting a protective role on the regulation of inflammatory responses.

## Figures and Tables

**Table 1:** Results of two-way ANOVA calculated for metabolites altered under bloodstream infection (BSI) and quantitated by ^1^H NMR spectroscopy. The metabolites included were derived from PCA.

	Metabolite	Mean ± SD			Treatment *p*-Value
		CON + TPN	SE + TPN	SE + COL	
**Plasma**	Lactate	1.46 ± 0.52 ^a^	6.00 ± 0.63 ^b^	1.32 ± 0.46 ^a^	<0.001
	Alanine	0.33 ± 0.05 ^ab^	0.43 ± 0.06 ^b^	0.22 ± 0.04 ^a^	0.046
	Succinate/pyruvate	0.02 ± 0.01 ^a^	0.08 ± 0.01 ^b^	0.01 ± 0.01 ^a^	0.001
	Choline	0.03 ± 0.01 ^a^	0.06 ± 0.01 ^b^	0.04 ± 0.00 ^ab^	0.021
	Glutamate	1.15 ± 0.16 ^a^	1.05 ± 0.19 ^a^	1.00 ± 0.14 ^a^	N.S. *
	*myo*-Inositol	8.35 ± 1.26 ^a^	8.66 ± 1.51 ^a^	8.56 ± 1.11 ^a^	N.S.
	Methionine	0.57 ± 0.06 ^b^	0.58 ± 0.07 ^b^	0.24 ± 0.05 ^a^	0.001
	Valine	0.20 ± 0.03 ^a^	0.26 ± 0.03 ^a^	0.37 ± 0.03 ^b^	0.001
	Leucine	0.02 ± 0.01 ^a^	0.03 ± 0.01 ^a^	0.07 ± 0.00 ^b^	<0.001
**CSF**	Lactate	0.34 ± 0.09 ^a^	0.62 ± 0.08 ^b^	0.30 ± 0.08 ^a^	0.021
	Alanine	0.01 ± 0.00 ^a^	0.02 ± 0.00 ^a^	0.01 ± 0.00 ^a^	0.066
	Choline	0.004 ± 0.001 ^a^	0.004 ± 0.001 ^a^	0.004 ± 0.001 ^a^	N.S.
	Succinate/pyruvate	0.03 ± 0.00 ^a^	0.02 ± 0.00 ^a^	0.02 ± 0.00 ^a^	0.064
	Glutamate	0.13 ± 0.01 ^a^	0.1 ± 0.01 ^a^	0.09 ± 0.01 ^a^	N.S.
	*myo*-Inositol	0.56 ± 0.06 ^a^	0.69 ± 0.05 ^a^	0.67 ± 0.05 ^a^	N.S.
	Methionine	0.004 ± 0.00 ^b^	0.003 ± 0.00 ^b^	0.001 ± 0.00 ^a^	<0.001
**PVMW**	Choline	4.83 ± 2.06 ^a^	0.85 ± 2.46 ^a^	8.54 ± 1.74 ^a^	0.060
	Lactate	8.44 ± 3.09 ^a^	2.10 ± 3.70 ^a^	12.66 ± 2.62 ^a^	0.095
	N-acetylaspartate	8.09 ± 3.10 ^a^	1.84 ± 3.70 ^a^	12.84 ± 2.62 ^a^	0.079
	Creatine	7.45 ± 3.34 ^a^	1.67 ± 3.99 ^a^	13.79 ± 2.82 ^a^	0.066

Values are represented as means ± standard deviation (SD). For blood and CSF, identified metabolites were quantified based on the known internal sodium trimethylsilyl-[2,2,3,3-^2^H_4_]-1-propionate (TSP) concentration and are expressed in mM. Brain metabolites were normalized to the total sum area under the peak prior to ANOVA analysis. The number of samples for each treatment group is as follows—for CSF samples: CON + TPN (*n* = 7), SE + TPN (*n* = 9), and SE + COL (*n* = 10); for plasma samples: CON + TPN (*n* = 7), SE + TPN (*n* = 5), and SE + COL (*n* = 9); and for brain samples: CON + TPN (*n* = 7), SE + TPN (*n* = 5), and SE + COL (*n* = 10). PVMW: periventricular white matter. * N.S. non-significant (*p* > 0.05). However, *p*-values below 0.1 are represented. Different subscript letters indicate statistically significant pair-wise differences of treatment.

## Supplementary Materials

The following are available online at http://www.mdpi.com/2218-1989/9/1/13/s1, Table S1. Litter and treatment × litter *p*-values of two-way ANOVA calculated for metabolites identified by PCA to be altered under bloodstream infection (BSI); Figure S1. PCA (a) score, and (b) loading plots of the NMR data of brain PVMW tissues. CON + TPN (green diamond) represents the control group, SE + TPN (red circles) represents the group infected by *Staphylococcus epidermidis*, and SE + COL (blue squares) represents the colostrum supplementation group. In the loading plots: Cho: choline; Lac: lactate; NAA: N-acetyl aspartate. L2, L3, and L4 are the second, third, and fourth loadings, respectively.
