# Supplementary material for: Rapid Cerebral Metabolic Shift during Neonatal Sepsis Is Attenuated by Enteral Colostrum Supplementation in Preterm Pigs
Source: Metabolites. 2019 Jan 11;9(1):13. doi: 10.3390/metabo9010013 (PMC6359096; doi:10.3390/metabo9010013)
Supplement: Supplementary file 1 [file metabolites-09-00013-s001.pdf]

Supplementary materials:

Table S1. Litter and treatment × litter p-values of two-way ANOVA calculated for metabolites identified by PCA to be altered under bloodstream infection (BSI).

	Metabolite	Litter P-value	Treatment *Litter P-value
**Plasma**	Lactate	0.007	<0.001
	Alanine	N.S.	N.S.
	Succinate / pyruvate	0.007	0.001
	Choline	N.S.	0.01
	Glutamate	N.S.	0.004
	myo-Inositol	N.S.	N.S.
	Methionine	0.009	N.S.
	Valine	N.S.	N.S.
	Leucine	N.S.	N.S.
**CSF**	Lactate	N.S.	N.S.
	Alanine	N.S.	N.S.
	Choline	N.S.	N.S.
	Succinate/ pyruvate	N.S.	0.013
	Glutamate	N.S.	N.S.
	myo-Inositol	N.S.	N.S.
	Methionine	N.S.	N.S.
**PVMW**	Choline	N.S.	N.S.
	Lactate	N.S.	N.S.
	N-acetylaspartate	N.S.	N.S.
	Creatine	N.S.	N.S.

A two-way ANOVA for an unbalanced design with the fixed effect of treatment, litter and treatment × litter was used to calculate the significance differences in metabolite concentrations of plasma and CSF, which were quantified based on the known internal TSP concentration and have the unit of mM, as well as brain metabolites which were normalized to total sum area under the peak prior to ANOVA analysis. Number of samples for each treatment group is as follows, for CSF samples: CON + TPN (n=7), SE + TPN (n=9), and SE + COL (n=10), plasma samples: CON + TPN (n=7), SE + TPN (n=5), and SE + COL (n=9) and brain samples: CON + TPN (n=7), SE + TPN (n=5), and SE + COL (n=10). Number of samples for each litter group is as follows, for CSF samples: litter 1 (n=13) and litter 2 (n=13), plasma samples: litter 1 (n=11) and litter 2 (n=10) and brain samples: litter 1 (n=12) and litter 2 (n=10). PVMW: periventricular white matter.

* N.S. non-significant (p>0.05).

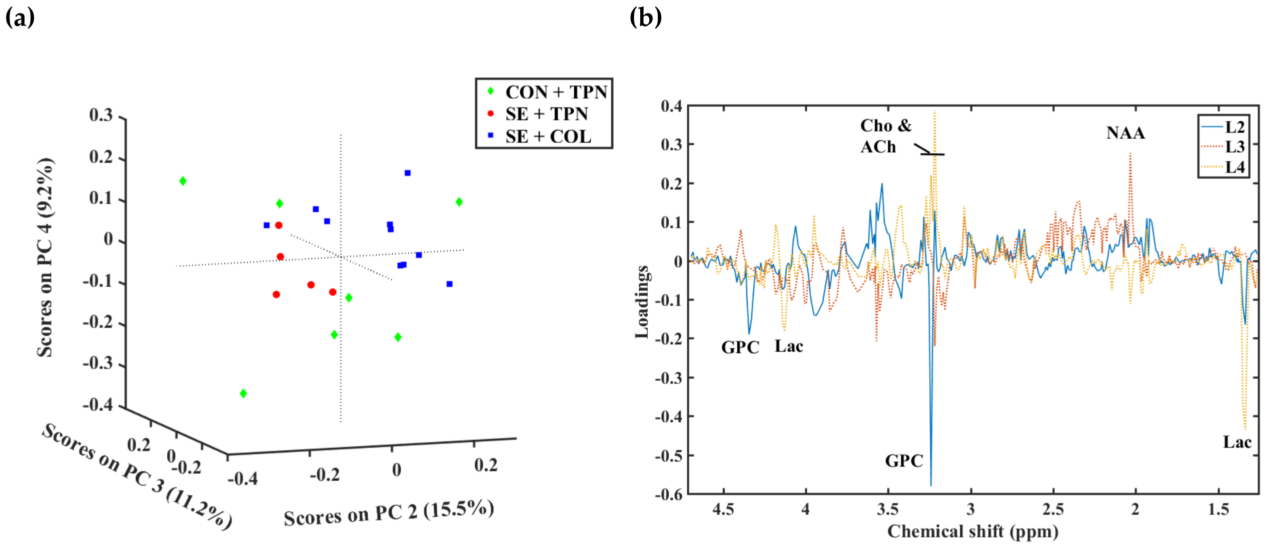

Figure S1. PCA (a) score, and (b) loading plots of the NMR data of brain PVMW tissues. CON + TPN (green diamond) represents the control group, SE + TPN (red circles) represents the group infected by *Staphylococcus epidermidis* and SE + COL (blue squares) represents the colostrum supplementation group. In the loading plots: Cho: choline; Lac: lactate; NAA: N-acetyl aspartate. L2, L3 and L4 are the second, third and fourth loadings, respectively.
